# Novel use of placental growth hormone in managing intrauterine growth restriction in Noonan syndrome

**DOI:** 10.1097/MS9.0000000000004143

**Published:** 2025-10-17

**Authors:** Iqra Akhtar, Maryum Tahir, Muddassir Khalid, Mohammed Hammad Jaber Amin

**Affiliations:** a Department of Medicine, Ayub Medical College, Abbottabad, Pakistan; b Department of Medicine, Nishtar Medical University, Multan, Pakistan; c Department of Medicine, Alzaiem Alazhari University, Khartoum, Sudan

*Dear Editor,*

I read a detailed study published in August 2025 that explores placental growth hormone as a potential treatment for intrauterine growth restriction in Noonan syndrome. The case study focuses on placental dysfunction and its rare genetic basis. Further clinical studies will be needed to confirm the effectiveness of this approach. A critical area of prenatal medicine is the convergence of new treatments and rare genetic disorders. Placental growth hormone polymorphisms and IUGR in Noonan syndrome have recently been linked to promising treatment options that could enhance pregnancy outcomes. One in 1000–2500 babies is affected by Noonan syndrome, which is caused by germline mutations in genes that encode proteins in the RAS/mitogen-activated protein kinase (MAPK) signaling pathway. Mutations in more than 20 genes cause this syndrome, though almost half the cases are due to mutations in PTPN11. Hyperactivation of the RAS/MAPK pathway interferes with key functions like metabolism, differentiation, and proliferation, causing intrauterine and postnatal growth failure^[1,2]^.

IUGR with Noonan syndrome in pregnancy is a complex clinical scenario. The majority of research has focused on growth retardation after birth; however, recent studies indicate that the dysregulated RAS/MAPK pathway affects fetal growth in numerous ways. This condition presents a treatment challenge, as it is associated with placental dysfunction, which serves as a crucial interface for fetal growth^[1,2]^. Placental insufficiency is responsible for approximately 75% of cases of IUGR. Another key regulatory hormone is human placental lactogen, which is secreted by the growth hormone variant gene on chromosome 17. From the fifteenth to the twentieth week, the pulsatile growth hormone secretion is replaced by PGH, expressed primarily in the syncytiotrophoblast and extravillous cytotrophoblast layers, in contrast to growth hormone^[3,4]^. In addition to gluconeogenesis, lipolysis, and anabolism in maternal tissues, PGH has a direct effect on fetal growth and placental development. PGH is expressed in extravillous trophoblasts and primarily regulates maternal insulin-like growth factor-I (IGF-I) levels. Research has shown that IUGR patients have lower placental synthesis of PGH, IGF-I, IGF-binding protein-1, and maternal serum PGH concentrations in pregnancies with fetal growth restriction^[4]^. A strong biological basis for targeted intervention is provided by the regulation of placental growth hormone and disruption of the RAS/MAPK pathway. The hallmark partial growth hormone insensitivity of Noonan syndrome, particularly in patients with PTPN11 variants, is caused by abnormal post-receptor signaling resulting from SHP2 overexpression^[1,2]^.

Growth hormone—IGF-1 is an essential regulator of fetal growth, and its abnormalities play a significant role in the pathophysiology of IUGR. Recent studies have shown that the downregulation of key regulatory proteins, such as FGF23 and Klotho, interferes with the GH/IGF-1/PI3K/AKT signaling pathway, leading to reduced intrauterine growth^[5]^. In animal studies, placental-targeted gene therapy has shown benefits. Human IGF-1 delivered to the placenta via nanoparticles regulates normal fetal growth and increases fetal glucose and placental nutrient transporter levels. Preclinical studies have demonstrated that the effects of placental growth hormone vary depending on the mode of administration; pulsatile treatment yields better growth benefits and fewer side effects compared to continuous use. This suggests that there may be room for more efficient administration. All of these findings support the use of placental growth hormone variations in clinical trials to treat IUGR in Noonan syndrome, shifting the focus from reactive postnatal care to proactive prenatal care. Due to genotype-specific variations in growth outcomes and treatment responses, genetic stratification based on particular Noonan syndrome variants is a crucial component of trial design.

PTPN11, RAF1, and KRAS mutations may be the best candidates to consider when targeting the first population, as patients in this group have more significantly slowed growth^[1,2]^.

TITAN Guidelines: This manuscript complies with TITAN Guidelines, 2025, declaring no use of AI^[6]^.

## Data Availability

Not applicable.

## Peer and provenance statement

Not commissioned and externally peer reviewed.
